# Automated segmentation of articular disc of the temporomandibular joint on magnetic resonance images using deep learning

**DOI:** 10.1038/s41598-021-04354-w

**Published:** 2022-01-07

**Authors:** Shota Ito, Yuichi Mine, Yuki Yoshimi, Saori Takeda, Akari Tanaka, Azusa Onishi, Tzu-Yu Peng, Takashi Nakamoto, Toshikazu Nagasaki, Naoya Kakimoto, Takeshi Murayama, Kotaro Tanimoto

**Affiliations:** 1 Department of Orthodontics and Craniofacial Development Biology, Graduate School of Biomedical and Health Sciences, Hiroshima University, Hiroshima, 734-8553 Japan; 2 Department of Medical System Engineering, Graduate School of Biomedical and Health Sciences, Hiroshima University, Hiroshima, 734-8553 Japan; 3 School of Dentistry, College of Dentistry, China Medical University, Taichung, 404 Taiwan; 4 School of Dentistry, College of Oral Medicine, Taipei Medical University, Taipei, 11031 Taiwan; 5 Department of Oral and Maxillofacial Radiology, Graduate School of Biomedical and Health Sciences, Hiroshima University, Hiroshima, 734-8553 Japan

**Keywords:** Dentistry, Diagnosis, Medical imaging, Magnetic resonance imaging

## Abstract

Temporomandibular disorders are typically accompanied by a number of clinical manifestations that involve pain and dysfunction of the masticatory muscles and temporomandibular joint. The most important subgroup of articular abnormalities in patients with temporomandibular disorders includes patients with different forms of articular disc displacement and deformation. Here, we propose a fully automated articular disc detection and segmentation system to support the diagnosis of temporomandibular disorder on magnetic resonance imaging. This system uses deep learning-based semantic segmentation approaches. The study included a total of 217 magnetic resonance images from 10 patients with anterior displacement of the articular disc and 10 healthy control subjects with normal articular discs. These images were used to evaluate three deep learning-based semantic segmentation approaches: our proposed convolutional neural network encoder-decoder named 3DiscNet (Detection for Displaced articular DISC using convolutional neural NETwork), U-Net, and SegNet-Basic. Of the three algorithms, 3DiscNet and SegNet-Basic showed comparably good metrics (Dice coefficient, sensitivity, and positive predictive value). This study provides a proof-of-concept for a fully automated deep learning-based segmentation methodology for articular discs on magnetic resonance images, and obtained promising initial results, indicating that the method could potentially be used in clinical practice for the assessment of temporomandibular disorders.

## Introduction

Temporomandibular disorder (TMD) is a collective term covering a number of clinical manifestations that involve pain and dysfunction of the masticatory muscles and temporomandibular joint (TMJ)^1^. The most common signs and symptoms of TMD are regional pain in the face and preauricular area, malocclusion, limited range of jaw movement, and TMJ noises and locking^2^.

According to a prospective cohort study of adults in the United States, the estimated annual incidence rate of first‐onset TMD is 3.9%, and it is typically accompanied with mild to moderate levels of pain and disability^3^. In developed countries, it is considered a widespread disorder affecting 5–12% of the population^4^.

Magnetic resonance (MR) imaging (MRI) is recognized as the best imaging modality for assessment of TMJ because it allows visualization of the anatomical and pathological features of all joint components^5^. Notably, MRI permits evaluation of the morphology and position of the articular disc, the presence or absence of reduction during mouth or jaw opening, the morphology and surface characteristics of the mandibular condyle, abnormal bone marrow signal in mandible and temporal bone, and the presence or absence of joint effusion. The most important subgroup of articular abnormalities in patients with TMD includes those with displacement and deformation of the articular disc^6^; this is an intracapsular disorder involving the disc-condylar complex, with a prevalence of 30–60% in patients with TMD^7^. Importantly, an MRI examination is expected for confirmation of the displacement and deformation of the articular disc, to ensure accurate diagnosis and prediction of treatment response.

Articular disc displacements can be subdivided into those with and without reduction^7^. Disc displacement with reduction is the most frequent type, and is characterized by the displaced disc returning to the normal position on mouth opening. In disc displacement without reduction, the articular disc is in a displaced position in relation to the superior part of the condyle in both closed- and open-mouth positions. In patients with articular disc displacement, the articular discs may be displaced when the mouth is open, or they may return to the correct position resulting in a significant change in the position of the disc between closing and opening. Therefore, a comprehensive diagnosis using both closed- and open-mouth MR images is necessary.

Artificial intelligence (AI) is gaining attention in various clinical disciplines and the dental field is no exception, with AI-based applications having been studied to streamline dental and oral care and improve the health of more people at a low cost^8–12^. Such AI-based applications should free dental professionals from time-consuming routine tasks and ultimately promote personalized, predictive, preventive, and participatory dental care^13^. Among the variety of AI algorithms available, the convolutional neural network (CNN)-based deep learning approach has become popular because of its excellent ability for object recognition when applied to medical images. Moreover, the increase in computational power and the pervasiveness of open-source frameworks have dramatically facilitated the development of CNNs^14^. In these circumstances, deep learning has been widely implemented for detection and segmentation purposes, and has showed encouraging performance. The fully convolutional network—a derivative form of the CNN—is the most widely used deep neural network in medical image segmentation, and several variants have been reported, including U-Net^15^ and SegNet^16^ architectures.

The aim of this study was to construct deep learning-based semantic segmentation algorithms for automatic detection and segmentation of the articular disc of the TMJ on MR images. The null hypothesis tested is that there is no difference in the performance of the three deep learning-based semantic segmentation algorithms for extracting articular discs from MR images.

## Materials and methods

### Dataset

This nonrandomized retrospective study was approved by the Ethical Committee for Epidemiology of Hiroshima University (Approval Number: E-2119). All methods in this study were performed in accordance with the Ethical Guidelines for Medical and Human Research Involving Human Subjects, Japan. Because of the retrospective design of this study, the requirement for informed consent was waived by the Ethical Committee for Epidemiology of Hiroshima University. The study included MR images of 10 patients with anterior displacement of the articular disc aged between 19 and 39 years (mean age of 26.4; 8 women, 2 men), and 10 healthy control subjects aged between 18 and 41 years (mean age of 27; 8 women, 2 men). Each subject underwent MRI on an Ingenia 3.0-T CX Quasar Dual scanner (Philips Healthcare, Best, the Netherlands). Only proton density-weighted sagittal images were used in this study. In total, 217 proton density-weighted sagittal images were used; 106 from the 10 patients and 111 from the 10 control subjects. These images included the left and right TMJ regions in mouth closed (102 images in total, 51 of patients and 61 of controls) and mouth open (115 images in total, 55 of patients and 60 of controls) positions. The patients with anterior displacement of the articular disc included those both with and without reduction. The number of consecutive MRI slices of the TMJ region in which the experts were able to visualize the articular disc differed between patients and healthy subjects, which is the reason why the number of included images differs between the two subject groups.

**Figure 1:**
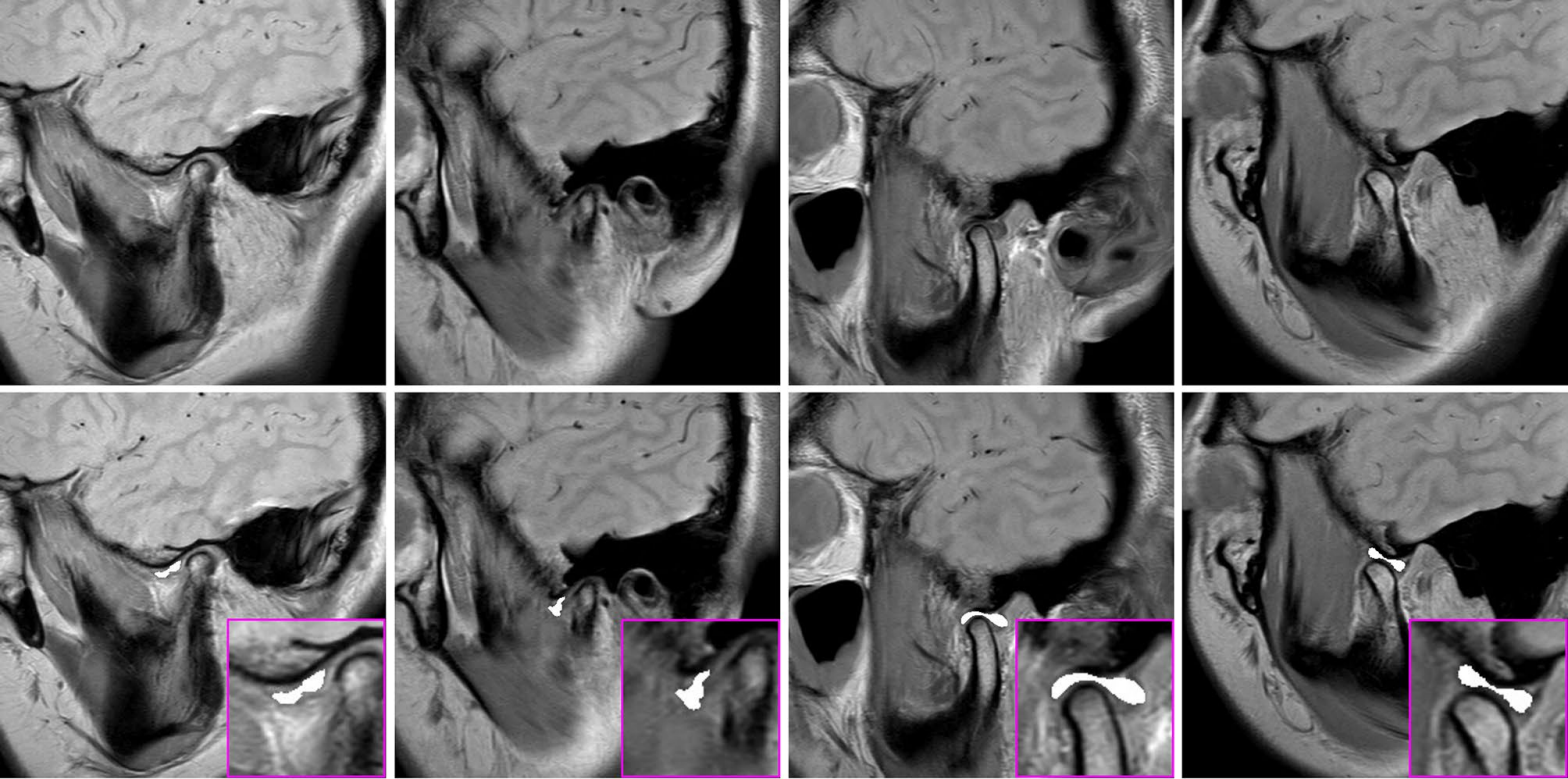
Representative images of manually segmented anterior discs. The first row shows raw MR images. The second row shows images with segmentations manually drawn by experts (white regions).

Two expert orthodontists (12 and 6 years of experience) and one expert oral and maxillofacial radiologist (25 years of experience) independently identified and manually segmented all articular discs of the TMJ on the MR images using ImageJ software (version 1.53, National Institutes of Health, Bethesda, MD; Fig. 1). The original MR data, saved in DICOM, were loaded into ImageJ and converted to JPEG files. The experts manually segmented the articular discs directly on a graphic tablet using a stylus pen (Artist 12, XP-PEN, Shenzhen, China). The articular disc region of the JPEG image was then filled in white (RGB value: 255, 255, 255), pixel-by-pixel, using the ‘pencil tool’ of Image J. Digital zooming was used to adjust the resolution as needed. After segmentation, the results were reviewed by three experts skilled in reading the articular disc region on MR images, and those images for which the experts were in agreement were adopted as the dataset. The manually segmented MR images (manual segmentation) were split into a training data set (80%) and test set (20%) for use in each of the following experiments. To derive a dataset showing the normal position of articular discs, the 111 images from the 10 control subjects were randomly split into 88 training images and 23 test images. For a dataset showing displaced articular disc positions, the 106 images from 10 patients were randomly split into 84 training images and 22 test images. For a dataset showing a mix of normal position and displaced articular discs, the 217 images were randomly split into 173 training images and 44 test images.

### Deep learning algorithms

All procedures were performed using an Intel Core i7-9750H 2.60-GHz central processing unit (Intel, Santa Clara, CA), 16.0 GB random access memory, and an NVIDIA GeForce RTX 2070 MAX-Q 8.0-GB graphics processing unit (NVIDIA, Santa Clara, CA). Deep learning algorithms were constructed using Python^17^ and were implemented using the Keras framework for deep learning with TensorFlow as the backend.

**Figure 2:**
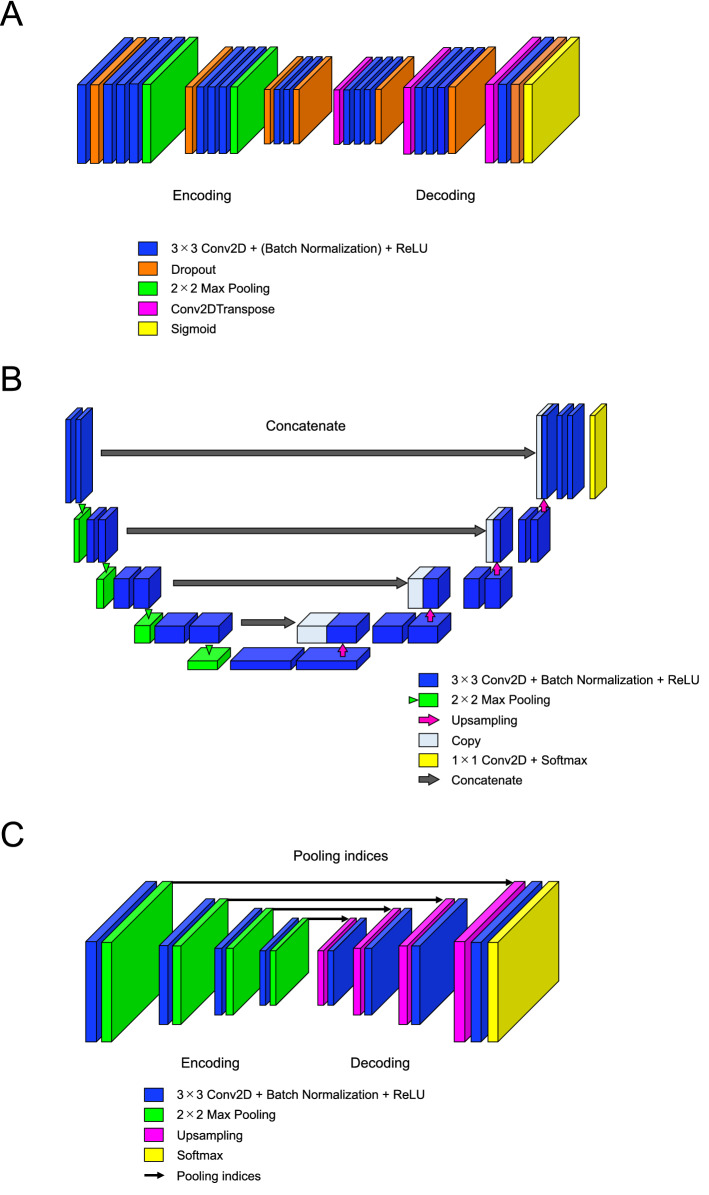
The encoder-decoder architecture of the deep learning models. (**A**) 3DiscNet, (**B**) original U-Net, and (**C**) original SegNet-Basic.

We adapted three convolutional semantic segmentation approaches: an encoder-decoder CNN, U-Net^15^, and SegNet^16^, which are all well suited to segmentation tasks. The overall architectures are shown in Fig. 2. In this study, we propose an encoder-decoder CNN model that we named 3DiscNet (Detection for Displaced articular DISC using convolutional neural NETwork), which has an asymmetric encoder-decoder architecture for the extraction of features at different spatial fields of view (Fig. 2A). To reduce the overfitting of the network, the dropout layer is placed behind the convolutional layers and max-pooling layers^18^. All the dropouts were given rates of 0.3 for the work described in this study. The final layer consists of a Sigmoid activation function that classifies each pixel as articular disc or background. The U-Net was a fully connected convolutional network that consists of convolution and max-pooling layers in the encoder part, and convolution and transpose layers in the decoder part. Encoder outputs were concatenated to the decoding layers to share spatial cues and to propagate the loss efficiently. The SegNet used a classical architecture for semantic pixel-wise segmentation, with encoder layers using max-pooling indices to upsample the feature maps and convolve them with a trainable decoder network. The original architectures of U-Net and SegNet are illustrated in Fig. 2B, C. The type of SegNet architecture used is currently termed SegNet-Basic^19^. The final layers are similar to the 3DiscNet, employing a sigmoid classifier instead of the original soft-max classifier in U-Net and SegNet-Basic. The U-Net and SegNet have shown promise for semantic segmentation of organs and pathology on MR images^20–22^.

**Figure 3:**
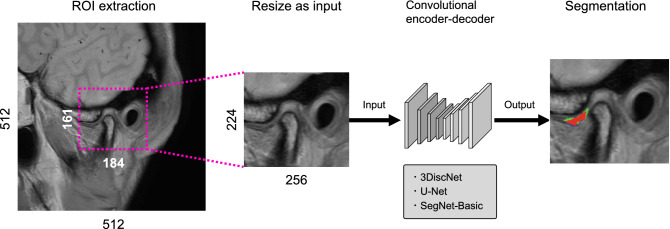
Schematic overview of data processing and articular disc segmentation with deep learning. MR images (512 × 512 pixels) are cropped into ROIs (161 × 184 pixels) and resized as patches (224 × 256 pixels) for use as input data. Patches are input to the three convolutional encoder-decoder networks. The segmentation results are shown as: Red, correct segmentation results; Blue, under-segmented regions; Green, over-segmented regions.

First, regions of interest (ROIs) around the articular discs were extracted from the datasets. The original image resolution was 512 × 512 pixels, and the ROIs, which were defined using a 161 × 184 pixel bounding box, were automatically cropped from the images using Python algorithms. The ROI images were then resized to 224 × 256 pixels for input into the three types of convolutional encoder-decoder network (Fig. 3). The 3DiscNet was trained using the Adam optimizer with a learning rate of 1.0 × 10^–3^, and the three algorithms were trained for a total of 2000 epochs.

### Performance metrics

The test data were used to validate the accuracy and computational efficacy of the models. The convolutional encoder-decoder network performance was assessed using the Dice similarity coefficient, sensitivity, and positive predictive value (PPV) of the test dataset. The Dice similarity coefficient, which is a popular similarity metric, was calculated using the following formula:

$$ Dice = \frac{{2\left| {P \cap T} \right|}}{\left| P \right| + \left| T \right|} $$

where *P* is the pixel area of the articular disc segmented with the convolutional encoder-decoder network, and *T* is the pixel area of the manually segmented ROI. The sensitivity is the percentage of the actual articular disc area correctly predicted as the articular disc area, defined as:

$$ Sensitivity = \frac{{\left| {P \cap T} \right|}}{\left| T \right|} $$

The PPV is a measure of the percentage of the correctly predicted articular disc area over the actual articular disc area as follows:

$$ PPV = \frac{{\left| {P \cap T} \right|}}{\left| P \right|} $$

A one-way analysis of variance (ANOVA) and post-hoc Tukey’s honestly significant difference (HSD) tests were used to analyze differences between the mean values of individual models. Statistical analyses were performed using IBM SPSS Statistics 27 (IBM Corp., Armonk, NY, USA).

## Results

**Figure 4:**
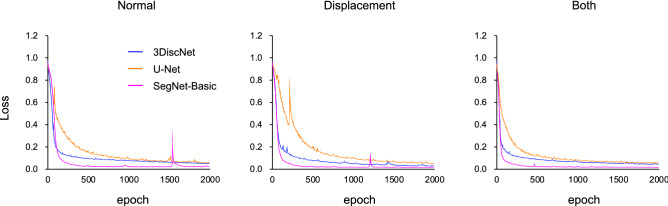
Training loss of each algorithm and dataset. (**A**) Dataset including normal articular disc images, (**B**) dataset including displaced articular disc images, and (**C**) dataset including both normal and displaced articular disc images.

**Figure 5:**
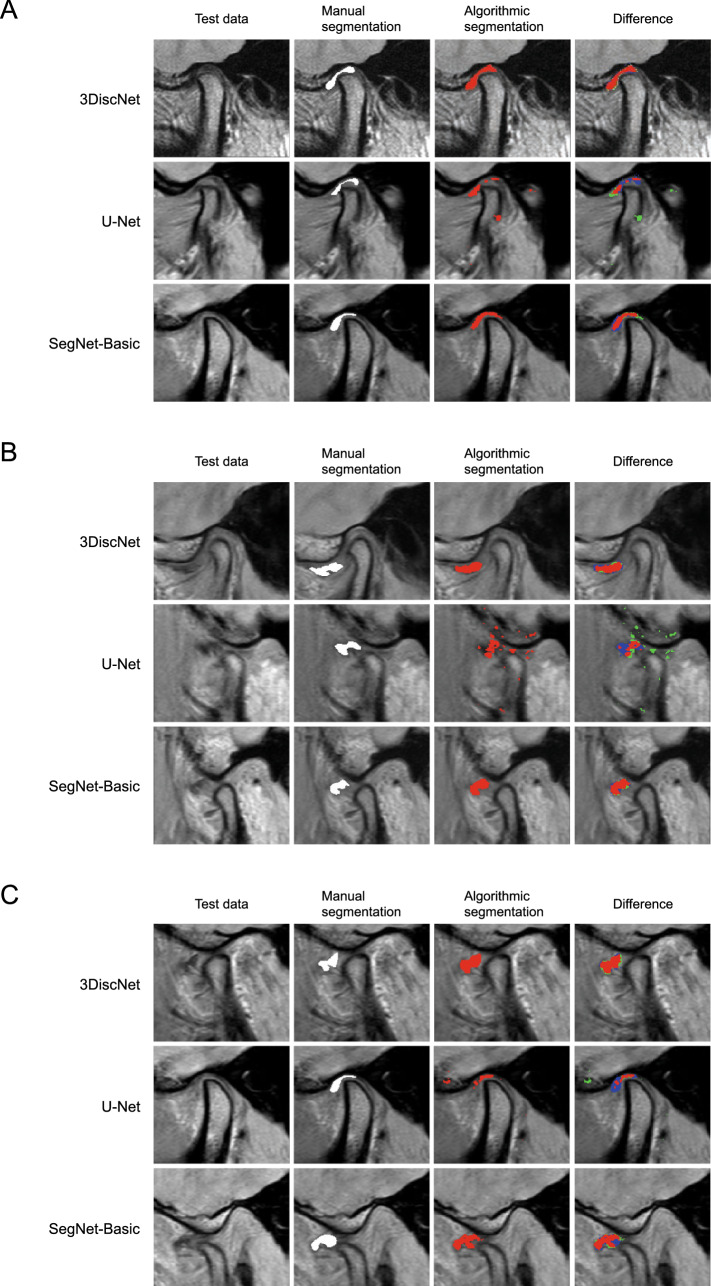
Representative segmentation results of the three deep learning algorithms. (**A**) Dataset including normal articular disc images, (**B**) dataset including displaced articular disc images, and (**C**) dataset including both normal and displaced articular disc images. The segmentation results are shown as: Red, correct segmentation results; Blue, under-segmented regions; Green, over-segmented regions.

As shown in Fig. 4, the training loss of each of the models decreased and converged, which indicates that these models did not show overfitting. The training loss dropped faster with the 3DiscNet model than with the U-Net and SegNet-Basic models, indicating faster convergence. Figure 5 shows representative examples of visual segmentation. The first column shows test data for validating algorithm performance, the second column shows manual segmentations, and the third and fourth columns show the algorithmic segmentations. Each row denotes a particular algorithm: 3DiscNet, U-Net, and SegNet-Basic, from the top downwards. Results obtained on the dataset containing only normal articular disc placement are shown in Fig. 5A; 3DiscNet and SegNet-Basic made predictions that were in good agreement with the manual segmentation data. Figure 5B shows the results for the dataset containing only patients with articular disc displacement. The results are similar to those shown in Fig. 5A, with 3DiscNet and SegNet-Basic making predictions that were in good agreement with the manual segmentation data. Figure 5C shows results for both normal and displaced articular discs; 3DiscNet and SegNet-Basic again made segmentations that were in good agreement with the manual segmentation data. However, the U-Net results showed a large number of false positives and false negatives with all of the datasets (Fig. 5).

**Figure 6:**
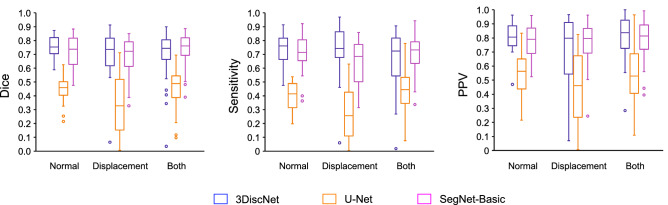
Boxplots of (**A**) Dice coefficient, (**B**) sensitivity, and (**C**) PPV distribution of each algorithm and dataset. Each circle represents outlier values.

We used the test data to evaluate the performance of the algorithms according to the three quantitative metrics of Dice coefficient, sensitivity, and PPV, computing the values for both normal and displaced articular disc segmentations. To reveal their distributions, these metrics are shown as box plots in Fig. 6 for 3DiscNet, U-Net, and SegNet-Basic, and for both normal and displacement test images. The Dice coefficient for segmentation performance was highest for SegNet-Basic, with the highest median accuracy and a small standard deviation in the dataset including both normal position and displaced articular discs. U-Net showed significantly lower values (and large standard deviations) than 3DiscNet and SegNet-Basic (one-way ANOVA with Tukey HSD; *p* < 0.001) for all three metrics, and the results were unstable for both normal position and displaced articular discs (Table 1).

**Table 1:** Performance metrics (mean ± standard deviation) of the three models.

Dataset	Model	Dice	Sensitivity	PPV
Normal	3DiscNet	**0.76 ± 0.08**^**a**^	**0.73 ± 0.12**^**a**^	**0.81 ± 0.11**^**a**^
	U-Net	0.45 ± 0.10^b^	0.40 ± 0.11^b^	0.55 ± 0.16^b^
	SegNet-basic	0.72 ± 0.11^a^	0.70 ± 0.15^a^	0.78 ± 0.12^a^
Displacement	3DiscNet	**0.70 ± 0.17**^**a**^	**0.72 ± 0.19**^**a**^	0.72 ± 0.23^a^
	U-Net	0.33 ± 0.21^b^	0.28 ± 0.20^b^	0.44 ± 0.24^b^
	SegNet-basic	0.68 ± 0.14^a^	0.64 ± 0.15^a^	**0.76 ± 0.16**^**a**^
Both	3DiscNet	0.70 ± 0.17^a^	0.66 ± 0.20^a^	0.80 ± 0.14^a^
	U-Net	0.46 ± 0.14^b^	0.44 ± 0.15^b^	0.54 ± 0.19^b^
	SegNet-basic	**0.74 ± 0.12**^**a**^	**0.70 ± 0.14**^**a**^	**0.80 ± 0.13**^**a**^

Bold values indicate the highest score for each performance metric.

Different letters indicate statistically significant differences between the algorithms (*p* < 0.001, ANOVA with Tukey HSD). They can be observed for each dataset and for each metric.

## Discussion

TMJ disc disorders caused by articular disc displacement, deformation, perforation, and fibrosis are the most common pathological conditions in TMD. Although MRI can provide a definitive diagnosis of TMJ disc disorder, concerns have been raised about the reliability of MRI nterpretations^23^. Previous studies have revealed that uncalibrated observers, even experienced experts, are unable to make accurate MRI assessments of disc disorders; the interpretation of MRI of the TMJ typically shows poor reproducibility^24–26^. These studies concluded that more effort is needed to understand the changes detectable on MRI. In this study using MR images, we demonstrated that a deep learning-based semantic segmentation approach can be applied to the detection and segmentation of TMJ articular discs. Our overall results showed that two deep learning algorithms—3DiscNet and SegNet-Basic—performed good detection and segmentation, whereas the U-Net algorithm did not obtain satisfactory results. The mean Dice coefficients for the dataset with both normal placement and displaced articular discs were 0.70 and 0.74 for 3DiscNet and SegNet-Basic, respectively. These are important results in that they show that the models can not only detect the existence of articular discs, but can also successfully find the locations of articular discs, regardless of normal positioning or displacement. However, the performance of U-Net was relatively poor, and its mean Dice coefficient was only 0.46. Indeed, the segmentation by U-Net revealed over-segmentation with the inclusion of irrelevant regions in addition to the articular disc. Our results demonstrated significant differences between U-Net and the other two algorithms in the performance metrics evaluating the extraction of articular discs from MR images, supporting rejection of the null hypothesis.

The articular disc lacks a clear border on MR images and its position is often displaced in patients with TMD, and therefore a great deal of variation in the shape and position of the disk is found among patients. Similarly, the prostate has also been reported as an organ with fuzzy boundaries on MR images^27^. These conditions make it difficult to detect and segment the articular disc accurately. Although U-Net was originally proposed for the segmentation of biological images with a limited quantity of training data^15^, studies have reported that it has a tendency for less accurate segmentation of objects with fuzzy boundaries^27–29^. Specifically, on very challenging images, U-Net tends to over-segment, under-segment, make false predictions, and even completely miss the target objects^29^. A previous study aiming to achieve mandibular canal segmentation on cone beam computed tomography (CBCT) images reported that U-Net mis- and over-segmented the mandibular canal region^30^. These reports are in accord with our results showing that the performance of U-Net on articular disc detection and segmentation was poor, even though 3DiscNet and SegNet-Basic showed comparably good metrics (i.e., Dice coefficient, sensitivity, and PPV) for all datasets.

SegNet was originally proposed for outdoor and indoor scene segmentation at a pixel level^16^. Some studies compared SegNet and U-Net for tissue segmentation, including that of Liu et al., who reported that SegNet showed more favorable performance than U-Net for cartilage and bone segmentation on musculoskeletal MR images^31^. Kwak et al. used SegNet and U-Net to segment the mandibular canal on the CBCT images of 102 patients, and also obtained good performance with SegNet^30^. However, to the contrary, Zhang et al. found that U-Net performed more favorable segmentation than SegNet when applied to breast MR images, which play a crucial role in diagnosis and the screening of those at high-risk of breast cancer^20^. Therefore, the suitability of these two models depends on the specific segmentation task and dataset, and appropriate comparisons will continue to be required.

Research on the application of AI to TMD has recently been reported, although the studies are limited to the diagnosis of TMJ osteoarthritis (OA) using CBCT images. A group from Brazil developed a system using deep learning that allows the staging of bony changes in TMJ OA^32,33^. Lee et al. tried to develop a system to detect TMJ OA on sagittal CBCT images using a deep learning method for object detection^34^. Two studies reported by US groups successfully integrated high-resolution CBCT and biological markers from patients with TMJ OA, with one study performing staging of condylar morphology^35^ and the other diagnosing the status of the disease^36^. While all these studies used CBCT, we have shown that AI can also be applied to MRI for TMD diagnosis. Given the results to date, including those from our proposed algorithms, it can be expected that AI systems for the diagnostic imaging of TMJ will be further developed, and will contribute to establishing a comprehensive diagnostic system for the maxillofacial region.

Our study had several limitations. All MRI scans were acquired at a single institution, and our models do not account for variations in hardware implementation and scanning techniques across institutions, which may bias the results. To increase the model robustness, evaluation of our concepts with a multicenter dataset is desirable. Moreover, the ‘ground truth’ manual segmentation images that we used to train the algorithms were also created by a limited number of experts. We manually segmented the articular discs as carefully as possible and then agreed on the ‘ground truth’ segmentation. Given the difficulty in interpreting MRI of the TMJ region, which was part of the motivation for performing this study, we believe that the participation of further experts will allow us to build a more widely acceptable dataset. Another limitation is that our study only made comparisons between the three models. Although AI has much potential, no algorithm can perform well on all possible problems. Therefore, the successful use of AI requires a great deal of effort by human experts^37^. Further studies are needed to optimize the structure of the CNNs, including comparisons with other models. In future work, we will modify the SegNet and 3DiscNet to include segmentation of other TMJ components (e.g., effusion, osteophytes) within the framework, and they will be trained and tested using a multicenter study.

## Conclusion

We conclude that within the limitations of this study, 3DiscNet and SegNet-Basic trained on manually segmented MR images can segment TMJ articular discs on MR images. This study provides a proof-of-concept for a deep learning-based fully automated segmentation methodology for articular discs on MR images, and it obtained promising initial results, indicating that the methodology could potentially be used for the assessment of TMD in clinical practice.

## References

[1] List T, Jensen RH (2017). Temporomandibular disorders: Old ideas and new concepts. Cephalalgia.

[2] Scrivani SJ, Keith DA, Kaban LB (2008). Temporomandibular disorders. N. Engl. J. Med..

[3] Slade GD (2013). Signs and symptoms of first-onset TMD and sociodemographic predictors of its development: The OPPERA prospective cohort study. J. Pain.

[4] Stimmer H (2020). Lesions of the lateral pterygoid muscle-an overestimated reason for temporomandibular dysfunction: A 3T magnetic resonance imaging study. Int. J. Oral Maxillofac. Surg..

[5] Johnson M, Sreela LS, Mathew P, Prasad TS (2021). Actual applications of magnetic resonance imaging in dentomaxillofacial region. Oral Radiol..

[6] Larheim TA (1995). Current trends in temporomandibular joint imaging. Oral Surg. Oral Med Oral Pathol. Oral Radiol. Endod..

[7] Lei J, Yap AU, Li Y, Liu MQ, Fu KY (2020). Clinical protocol for managing acute disc displacement without reduction: A magnetic resonance imaging evaluation. Int. J. Oral. Maxillofac. Surg..

[8] Krois J (2019). Deep learning for the radiographic detection of periodontal bone loss. Sci. Rep..

[9] Mine Y, Suzuki S, Eguchi T, Murayama T (2020). Applying deep artificial neural network approach to maxillofacial prostheses coloration. J. Prosthodont. Res..

[10] Kuwana R (2021). Performance of deep learning object detection technology in the detection and diagnosis of maxillary sinus lesions on panoramic radiographs. Dentomaxillofac. Radiol..

[11] Yoo JH (2021). Deep learning based prediction of extraction difficulty for mandibular third molars. Sci. Rep..

[12] Takeda S, Mine Y, Yoshimi Y, Ito S, Tanimoto K, Murayama T (2020). Landmark annotation and mandibular lateral deviation analysis of posteroanterior cephalograms using a convolutional neural network. J. Dent. Sci..

[13] Schwendicke F, Samek W, Krois J (2020). Artificial intelligence in dentistry: Chances and challenges. J. Dent. Res..

[14] Desai AD (2021). The international workshop on osteoarthritis imaging knee MRI segmentation challenge: a multi-institute evaluation and analysis framework on a standardized dataset. Radiol. Artif. Intell..

[15] Ronneberger, O., Fischer, P. & Brox, T. U-net: Convolutional networks for biomedical image segmentation. In *Lecture Notes in Computer Science (including subseries Lecture Notes in Artificial Intelligence and Lecture Notes in Bioinformatics)* vol. 9351, 234–241 (Springer, 2015).

[16] Badrinarayanan, V., Handa, A., & Cipolla, R. Segnet: A deep convolutional encoder-decoder architecture for robust semantic pixel-wise labelling. Preprint at https://arxiv.org/abs/1505.07293 (2015).

[17] Python. https://www.python.org/.

[18] Srivastava N, Hinton G, Krizhevsky A, Sutskever I, Salakhutdinov R (2014). Dropout: A simple way to prevent neural networks from overfitting. J. Mach. Learn. Res..

[19] Badrinarayanan V, Kendall A, Cipolla R (2017). Segnet: A deep convolutional encoder-decoder architecture for image segmentation. IEEE Trans. Pattern Anal. Mach. Intell..

[20] Zhang L, Mohamed AA, Chai R, Guo Y, Zheng B, Wu S (2020). Automated deep learning method for whole-breast segmentation in diffusion-weighted breast MRI. J. Magn. Reson. Imaging.

[21] Wei J, Xia Y, Zhang Y (2019). M3Net: A multi-model, multi-size, and multi-view deep neural network for brain magnetic resonance image segmentation. Pattern Recognit..

[22] Grøvik E, Yi D, Iv M, Tong E, Rubin D, Zaharchuk G (2020). Deep learning enables automatic detection and segmentation of brain metastases on multisequence MRI. J. Magn. Reson. Imaging.

[23] Naeije M, Te Veldhuis AH, Te Veldhuis EC, Visscher CM, Lobbezoo F (2013). Disc displacement within the human temporomandibular joint: A systematic review of a 'noisy annoyance'. J. Oral Rehabil..

[24] Nebbe B, Brooks SL, Hatcher D, Hollender LG, Prasad NG, Major PW (2000). Magnetic resonance imaging of the temporomandibular joint: Interobserver agreement in subjective classification of disk status. Oral Surg. Oral Med Oral Pathol. Oral Radiol. Endod..

[25] Widmalm SE, Brooks SL, Sano T, Upton LG, McKay DC (2006). Limitation of the diagnostic value of MR images for diagnosing temporomandibular joint disorders. Dentomaxillofac. Radiol..

[26] Butzke KW, Batista Chaves KD, Dias da Silveira HE, Dias da Silveira HL (2010). Evaluation of the reproducibility in the interpretation of magnetic resonance images of the temporomandibular joint. Dentomaxillofac. Radiol..

[27] Zhu, Q., Du, B., Turkbey, B., Choyke, P. L. & Yan, P. Deeply-supervised CNN for prostate segmentation. In *International Joint Conference on Neural Networks IEEE* 178–184 (2017).

[28] Lee, H. J., Kim, J. U., Lee, S., Kim, H. G., & Ro, Y. M. Structure Boundary Preserving Segmentation for Medical Image with Ambiguous Boundary. In *Proceedings of the IEEE/CVF Conference on Computer Vision and Pattern Recognition* 4817–4826 (2020).

[29] Ibtehaz N, Rahman MS (2020). MultiResUNet : Rethinking the U-Net architecture for multimodal biomedical image segmentation. Neural Netw..

[30] Kwak GH (2020). Automatic mandibular canal detection using a deep convolutional neural network. Sci. Rep..

[31] Liu F, Zhou Z, Jang H, Samsonov A, Zhao G, Kijowski R (2018). Deep convolutional neural network and 3D deformable approach for tissue segmentation in musculoskeletal magnetic resonance imaging. Magn. Reson. Med..

[32] de Dumast P (2018). A web-based system for neural network based classification in temporomandibular joint osteoarthritis. Comput. Med. Imaging Graph..

[33] Ribera NT (2019). Shape variation analyzer: A classifier for temporomandibular joint damaged by osteoarthritis. Proc. SPIE Int. Soc. Opt. Eng..

[34] Lee KS (2020). Automated detection of TMJ osteoarthritis based on artificial intelligence. J. Dent. Res..

[35] Shoukri B (2019). Minimally invasive approach for diagnosing TMJ Osteoarthritis. J. Dent. Res..

[36] Bianchi J (2020). Osteoarthritis of the Temporomandibular Joint can be diagnosed earlier using biomarkers and machine learning. Sci. Rep..

[37] Waring J, Lindvall C, Umeton R (2020). Automated machine learning: Review of the state-of-the-art and opportunities for healthcare. Artif. Intell. Med..

